# Contribution of Elevated Glucose and Oxidized LDL to Macrophage Inflammation: A Role for PRAS40/Akt-Dependent Shedding of Soluble CD14

**DOI:** 10.3390/antiox12051083

**Published:** 2023-05-11

**Authors:** Lucía Sanjurjo, Esmeralda Castelblanco, Josep Julve, Nuria Villalmanzo, Érica Téllez, Anna Ramirez-Morros, Núria Alonso, Dídac Mauricio, Maria-Rosa Sarrias

**Affiliations:** 1 Innate Immunity Group, Health Sciences Research Institute Germans Trias i Pujol (IGTP), 08916 Badalona, Spain; l.sanxurxo@gmail.com (L.S.); etellez@igtp.cat (É.T.); 2 Division of Endocrinology, Metabolism and Lipid Research, Department of Internal Medicine, Washington University School of Medicine, St. Louis, MO 63110, USA; esmeralda@wustl.edu; 3 Unitat de Suport a la Recerca Barcelona, Institut Universitari d’Investigació en Atenció Primària Jordi Gol i Gurina, 08007 Barcelona, Spain; amramirez.cc.ics@gencat.cat (A.R.-M.); didacmauricio@gmail.com (D.M.); 4 Endocrinology, Diabetes and Nutrition Group, Institut de Recerca de l’Hospital de la Santa Creu i Sant Pau (IRHSCSP), 08041 Barcelona, Spain; jjulve@santpau.cat; 5 Centre for Biomedical Research on Diabetes and Associated Metabolic Diseases (CIBERDEM), ISCIII, 28029 Madrid, Spain; 6 Department of Endocrinology and Nutrition, Health Sciences Research Institute and University Hospital Germans Trias i Pujol, 08916 Badalona, Spain; nuriavillalmanzo@hotmail.com; 7 Gerència Territorial de la Catalunya Central, Institut Català de la Salut, 08272 Sant Fruitós de Bages, Spain; 8 Department of Endocrinology and Nutrition, Hospital de la Santa Creu i Sant Pau and Sant Pau Biomedical Research Institute, 08041 Barcelona, Spain; 9 Faculty of Medicine, University of Vic—Central University of Catalonia, 08500 Vic, Spain; 10 Centre for Biomedical Research on Liver and Digestive Diseases (CIBEREHD), ISCIII, 28029 Madrid, Spain

**Keywords:** diabetes, atherosclerosis, CD36, foam cell, monocytes, TLR4, vascular

## Abstract

Atherosclerosis, a process in which macrophages play a key role, is accelerated in diabetes. Elevated concentrations of serum-oxidized low-density lipoproteins (oxLDL) represent a common feature of both conditions. The main goal of this study was to determine the contribution of oxLDL to the inflammatory response of macrophages exposed to diabetic-mimicking conditions. THP1 cells and peripheral blood monocytes purified from non-diabetic healthy donors were cultured under normal (5 mM) or high glucose (HG) (15 mM) with oxLDL. Then, foam cell formation, expression of CD80, HLADR, CD23, CD206, and CD163, as well as toll-like receptor 4 (TLR4) and co-receptors CD36 and CD14 (both at the cell surface and soluble (sCD14)), and inflammatory mediators’ production were measured by flow cytometry, RT-qPCR, or ELISA. Additionally, serum sCD14 was determined in subjects with subclinical atherosclerosis with and without diabetes by ELISA. Our results showed that oxLDL-mediated intracellular lipid accumulation via CD36 increased under HG and that HG + oxLDL enhanced TNF, IL1B, and IL8, and decreased IL10. Moreover, TLR4 was upregulated in macrophages under HG and monocytes of subjects with diabetes and atherosclerosis. Interestingly, HG-oxLDL upregulated CD14 gene expression, although its total cellular protein abundance remained unaltered. sCD14 shedding via PRAS40/Akt-dependent mechanisms, with pro-inflammatory activity, was significantly increased in cultured macrophages and plasma from subjects with diabetes and subclinical atherosclerosis or hypercholesterolemia. Our data support an enhanced synergistic pro-inflammatory effect induced by HG and oxLDL in cultured human macrophages, possibly explained by increased sCD14 shedding.

## 1. Introduction

Atherosclerosis is a chronic inflammatory condition characterized by the accumulation of lipids and inflammatory cells in the vessel wall, and monocytes/macrophages play a major role in its development [1,2]. The course of atherosclerotic disease is accelerated in subjects with diabetes [3]. Consistently, the prevalence, incidence, and mortality from atherosclerotic cardiovascular diseases are increased in subjects with diabetes [4]. Histological analysis performed in atherosclerotic coronary plaques of type 2 diabetes mellitus (T2D) patients confirmed an accelerated course of atherosclerotic disease [5,6,7].

The precise pathogenic mechanisms underlying diabetes-accelerated atherosclerosis are still poorly defined. Accumulating evidence suggests that sustained hyperglycemia and chronic hyperinsulinemia negatively influence the biology of vascular cells, and hence contribute to the adverse structural and functional remodeling of the vasculature [3].

Apart from hyperglycemia and altered insulin signaling, the impact of atherogenic dyslipidemia is a common finding in many subjects with diabetes mellitus. In this regard, elevated values of circulating triglycerides, cholesterol remnants, and small-dense low-density lipoproteins (LDL), which in turn are more prone to be oxidized, have been described in subjects with T2D. Resulting oxidized LDL (oxLDL) are extremely reactive lipoproteins that are efficiently recognized by macrophage innate immune pattern recognition receptors (PRRs). As such, PRRs may facilitate the uptake of oxLDL, and hence promote lipid accumulation in macrophages. Foam cell formation and activation has been linked to vascular inflammation and plaque growth [8,9,10]. It is noteworthy that elevations in circulating oxLDL are a common hallmark of T2D subjects [11,12], and predict 10-year progression of subclinical atherosclerosis, independent of other quantitative characteristics (i.e., cholesterol content, the number, and the size) of LDL particles [13].

Scavenger receptors are the main PRRs involved in oxLDL-mediated recognition and uptake and inflammatory signaling in macrophages. Among them, CD36, an 88 kDa transmembrane glycoprotein expressed in many cell types, accounts for a large proportion of oxLDL uptake by macrophages [14]. Another key player in mediating pro-inflammatory oxLDL-mediated signaling are members of the toll-like receptor (TLRs) family (i.e., TLR2 and TLR4), a family of PRRs that respond to different inflammatory mediators (i.e., derived from pathogens or endogenous danger molecules, resulting in inflammatory activation) [15]. Notably, CD36 has also been shown to transduce signals that regulate oxLDL-dependent inflammatory responses in cooperation with TLRs [15]. TLRs were initially believed to have an autonomous recognition and transcriptional signaling capacity, but an increasing body of evidence suggests that accessory receptors such as CD36 or CD14 are necessary for efficient ligand binding and signaling [16]. In this regard, the inflammatory responses of macrophages to oxLDL depend on the cooperation of TLR4 and TLR6 with CD36 [17], which facilitates endocytosis and the intracellular conversion of those soluble ligands into crystals or fibrils, which results in lysosomal disruption and activation of the NLRP3 inflammasome [2].

Another widely recognized factor as a potent stimulator of monocyte activity is hyperglycemia associated with diabetes. In agreement, a more pro-inflammatory circulating monocyte phenotype with higher secretion levels of pro-inflammatory cytokines has been reported in cellular and animal models and in subjects with T1D and T2D compared with non-diabetic controls [18,19,20,21,22,23,24]. This pro-inflammatory profile appears to be due to increased TLR2 and TLR4 expression [24,25].

Among the different downstream pathways that regulate monocyte activation, the PI3K/Akt/mTOR axis is of key relevance in the context of metabolic disorders. Within this pathway, proline-rich Akt substrate 40 kDa (PRAS40) was originally identified as a novel substrate of Akt [26]. It bridges cell signaling between Akt and the mammalian target of rapamycin complex 1 (mTORC1). As a downstream target of Akt, PRAS40 functions as an integral component between Akt and mTOR and has important roles in the biological activity and pathogenesis of a variety of diseases, including diabetes [27,28]. Upon Akt activation by insulin, PRAS40 is phosphorylated, and released from mTOR; as a result, insulin-receptor substrate-1 (IRS-1) is inactivated, thereby reducing insulin sensitivity. Overall, this sequence of events may provide evidence for a potential role for PRAS40 in insulin resistance [28]. Regarding atherosclerosis, PRAS40 suppresses atherosclerosis via inhibition of endothelial mTORC1-mediated pro-inflammatory signaling [29].

However, despite existing evidence showing hyperglycemia- or oxLDL-mediated activation of immune cells, including macrophages, the receptor-mediated mechanisms of macrophage inflammatory responses by both hyperglycemic and atherogenic conditions have not yet been fully explored.

The purpose of the present study was to determine the effect of the combination of atherogenic and hyperglycemic conditions in human macrophages. Herein, we analyzed oxLDL-dependent macrophage foam cell formation, inflammatory responses, and the expression of key cellular components under hyperglycemic conditions in vitro and ex vivo using circulating monocytes isolated from T2D and subclinical atherosclerosis subjects. Our findings suggest a novel pro-inflammatory mechanism that involves CD14 shedding through enhanced Akt/PRAS40 activation, which may explain the increase in macrophage inflammatory responses in diabetes-accelerated atherosclerosis.

## 2. Materials and Methods

### 2.1. Reagents

Phosphate-buffered saline (PBS) consisted of 150 mM NaCl (S7653), 8 mM Na_2_HPO_4_ (NIST218611), and 1.5 mM KH_2_PO_4_ (NIST200B), pH 7.4. TBS consisted of 140 mM NaCl and 50 mM Tris-HCl (T5941), pH 7.4, and TBS lysis buffer consisted of 20 mM Tris, pH 7.5, containing 150 mM NaCl, 1 mM EDTA (EDS), 1 mM EGTA (E3889), 1% Triton X-100 (X100), 1 mM Na_3_VO_4_ (S6508), 1 mM PMSF (P7627), and complete protease inhibitor cocktail (P8340), all from Sigma-Aldrich (Merck KGaA, Darmstadt, Germany).

### 2.2. Primary Cells from Healthy Donors and Cell Lines

Buffy coats, provided by the Blood and Tissue Bank (Barcelona, Spain), were obtained from healthy blood donors following institutional standard operating procedures for blood donation and processing. All subjects had a normal fasting glucose level of 105.1 ± 36.2 mg/dL and glycosylated hemoglobin of 5.3% ± 0.2%. CD3^+^ cells were depleted using the RosetteSep human CD3 depletion cocktail (StemCell Technologies, Vancouver, BC, Canada, 15621). Peripheral blood mononuclear cells were isolated as previously described [30] by Ficoll–Paque (GE Healthcare, Chicago, IL, USA, 17–1440) density gradient centrifugation at 400× *g* for 25 min. Recovered cells were washed twice in PBS and counted using Perfect-Count Microspheres (Cytognos, Salamanca, Spain, CYT-PCM) following the manufacturer’s instructions. Peripheral blood (PB) monocytes were isolated by adhesion in culture dishes, by 30 min incubation in complete RPMI-1640 medium (Lonza, Basel, Switzerland, BE12-702F) containing 10% AB human serum (Sigma-Aldrich, H4522). Non-adherent cells were removed, and the adherent cells were washed twice with PBS and incubated in RPMI 10% heat-inactivated fetal calf serum (FCS, Lonza, DE14–840E), 100 μg/mL of penicillin, and 100 μg/mL of streptomycin (Sigma-Aldrich, P0781) for 24 h prior to the experiments. The percentage of adherent CD14^+^ cells routinely obtained was 94.98% (+/− 3.26%). In assays performed with human monocyte-derived macrophages (MDM), PB monocytes were differentiated by incubation in RPMI 10% FCS for 14 days prior to the experiments, as previously described [31]. Culturing MDM without any additional growth factor lowered the possibility of deriving polarized macrophages.

Human leukemia monocytic THP1 cell culture conditions have been previously described by Amézaga et al. [31]. Cells were grown in RPMI-1640, 100 μg/mL of penicillin, and 100 μg/mL of streptomycin, supplemented with 10% FCS. When indicated, cells were differentiated into macrophages (MΦ) prior to the experiments by incubation with 10 ng/mL of phorbol 12-myristate 13-acetate (PMA, Sigma-Aldrich, P8139) in culture medium for 24 h. They were then washed with PBS and grown in culture medium for 24 h. These cells are referred to as THP1 MΦ.

MDM and THP1 were incubated with 150 μg/mL of endotoxin-free oxidized low-density lipoprotein (oxLDL, Alfa Aesar, Haverhill, MA, USA, J65591) in RPMI medium, containing 5.5 mmol/L of glucose (normal glucose, NG) and 15 mmol/L of glucose (high glucose, HG, D-(+)-Glucose solution, Sigma-Aldrich, G8769), with daily supplementation of the medium. As an osmotic control, 15 mmol/L of mannitol (Applichem, Darmstadt, Germany, A4831) was added. When indicated, the cells were pretreated for 45 min with 10 μM of wortmannin (W, Invivogen, San Diego, CA, USA, tlr-wtm) or incubated with human recombinant soluble CD14 (rsCD14, Preprotech, London, UK, 110-01).

### 2.3. Human Samples from Subjects with and without Diabetes

Circulating sCD14 concentrations were determined in serum samples from subjects with (n = 69) and without (n = 70) T2D from two previously recruited cohorts (i.e., the outpatient clinics at University Hospital Arnau de Vilanova (Lleida) and outpatient clinics and asymptomatic subjects undergoing carotid surgical endarterectomy at University Hospital Germans Trias i Pujol (Badalona)). Moreover, serum sCD14 concentrations were also determined in subjects with and without diabetes from the outpatient clinics at University Hospital Germans Trias i Pujol, in which immunophenotyping studies were performed. A full description of these cohorts can be found in previous publications from our group [32,33,34]. All the study subjects were free from previous clinical atherosclerotic cardiovascular disease (CVD) (coronary heart disease, stroke, or peripheral vascular disease). The inclusion criteria were as follows: age > 18 years, and normal renal function (estimated glomerular filtration rate (eGFR) > 60 mL/min). We excluded subjects with a urine albumin excretion ratio > 300 mg/g. Furthermore, patients under current treatment with dipeptidyl peptidase 4 inhibitors or glucagon-like peptide-1 receptor agonists were excluded. Additionally, the non-diabetic control participants had fasting glucose and HbA1c values below 100 mg/dl and 5.7%, respectively. The clinical characteristics of the study group are summarized in Table 1. The Local Ethics Committee of the center approved the protocol, and all participants signed informed consent forms.

All study participants underwent the same ultrasound examination to analyze the presence or absence of atheromatous plaques. B-mode ultrasound and color Doppler examinations of carotid and femoral sites were performed using a LOGIQ^®^ E9—General Electric (GE, Healthcare, Waukesha, WI, USA) device equipped with a 15 MHz linear array probe, previously explained in [35]. The presence of atheromatous plaque was assessed in ten vascular territories: internal, bulb and common carotid, and common and superficial femoral arteries. Atheromatous plaque was defined as intima media thickness (cIMT) > 1.5 mm protruding into the lumen, according to the ASE Consensus Statement and the Mannheim cIMT Consensus [36].

### 2.4. Quantification of Foam Cell Formation

THP1 MΦ (2.5 × 10^5^ cells/well) were incubated with 150 μg/mL of oxLDL for 24 h in RPMI 1% FCS or medium alone in NG or HG conditions, and subsequently stained with Nile Red, as follows. Cells were fixed in 10% (*v*/*v*) formalin (Sigma-Aldrich, HT501128) for 1 h and incubated with a 1 mM Nile Red solution (Sigma-Aldrich, 19123) in DMSO. Next, the cells were extensively washed with cold PBS and harvested with accutase (Sigma-Aldrich, A6964). Nile Red incorporation was analyzed by flow cytometry on a FACSCantoII instrument (BD Biosciences, Franklin Lakes, NJ, USA) using FACSDiva software (BD Biosciences). Fold increase levels were calculated using the mean fluorescence intensity (MFI) values of untreated cells cultured in NG conditions as a reference.

### 2.5. DiI-oxLDL Uptake

THP1 MΦ (2.5 × 10^5^ cells/well) were incubated for 6 h at 37 °C with 5 μg/mL of Di-l-labeled oxLDL (Dil-oxLDL; Alfa-Aesar, J64164) in RPMI-1640 containing 0.2% (*v*/*v*) fatty acid-free BSA (Sigma-Aldrich, A7030) in NG or HG conditions. For competition analysis, a 40× molar excess of unlabeled oxLDL or human albumin (Alb, Sigma-Aldrich, A9731) was added to the medium 1 h before the addition of Dil-oxLDL. After washing with PBS, cells were harvested by accutase treatment (Sigma-Aldrich, A6964) and uptake was analyzed by flow cytometry on a FACSCantoII instrument (BD Bioscience) using FACSDiva software (BD Biosciences).

### 2.6. Measurement of mRNA Levels by Real-Time PCR (RT-PCR)

THP1 MΦ or MDM (10^6^ cells/well) were treated for 24 h in RPMI medium containing 5% FCS. Cells were washed with PBS and disrupted with QIAzol Lysis Reagent (Qiagen, Hilden, Germany 79306), and total RNA was extracted using the miRNeasy Mini Kit (Qiagen, 217004). Total RNA (1 μg) was reverse-transcribed using the Transcriptor First-Strand cDNA Synthesis Kit (Roche, Basilea, Switzerland, 04379012001). Each reaction was then amplified in a LightCycler^®^ 480 PCR system using KAPA SYBR Fast Master Mix (KAPA Biosystems, Woburn, MA, USA, 51230–100). Samples were incubated for an initial denaturation at 95 °C for 5 min, followed by 40 PCR cycles using the following conditions: 95 °C for 10 s, 60 °C for 20 s, and 72 °C for 10 s. The primer pairs used are summarized in Table 2. Gene expression values were normalized to the expression levels of glyceraldehyde 3-phosphate dehydrogenase (GAPDH). Fold induction levels were calculated using the levels of expression of each gene in the NG untreated condition as a reference.

### 2.7. Western Blot Analysis of Cell Lysates and Culture Supernatants

THP1 MΦ or MDM (10^6^ cells/well) were incubated with oxLDL in medium containing 5% FCS under HG or NG conditions for 24 h. Cells were then washed in cold TBS and lysed in lysis buffer for 30 min at 4 °C. Nuclei and cell debris were removed by centrifugation at 8000× *g* for 15 min, and the protein concentrations in cell lysates as well as in culture supernatants were measured with the BCA protein assay reagent kit (Thermo Fisher, Waltham, MA, USA 23227), following the manufacturer’s instructions. For secreted protein analysis, 1 mL of culture supernatant was precipitated in 14% TCA (Sigma-Aldrich, T6508) and 0.14% Triton X-100 (Sigma-Aldrich, X100) for 30 min at 4 °C. The proteins were pelleted at 12,000 rpm for 30 min at 4 °C and washed with cold acetone. Next, 40 to 50 μg of protein from cell lysates or 1 mL of precipitated supernatants were resolved in 10% SDS-polyacrylamide gels under reducing conditions and electrophoretically transferred to nitrocellulose membranes (Bio-Rad Laboratories, Hercules, CA, USA, 162-0115). After Ponceau S staining (Sigma-Aldrich, P3504), the membranes were blocked with Starting Block TBS buffer (Thermo Fisher Scientific Inc., Waltham, MA, USA, PI37542) for 1 h at room temperature and incubated overnight at 4 °C with the indicated antibodies: poAb anti-CD36 (Sigma-Aldrich, HPA002018), moAb anti-TLR4 (Novus Biologicals, Centennial, CO, USA, NB100-56566), poAb anti-CD14 (Sigma-Aldrich, HPA001887), poAb anti-phospho Akt (Ser473) (Cell Signaling, Danvers, MA, USA, 9271), and moAb phosho-PRAS40 (Thr246) (R&D systems, Minneapolis, MN, USA, MAB68901), all diluted in Starting Block TBS buffer. Blots were also probed with antibodies against actin (poAb anti-ACTA1, Sigma-Aldrich, A2066) or α-tubulin (mAb anti-TUBA4A; Sigma-Aldrich, T9026) to evaluate equal loading. The membranes were subsequently incubated with the appropriate fluorescently coupled secondary antibodies (IRDye680Cw-conjugated goat anti-rabbit IgG or IRDye 800Cw-conjugated goat anti-mouse IgG, LI-COR Biosciences, Lincoln, NE, USA, 926–32221 and 926–32210, respectively) diluted in Starting Block TBS buffer for 60 min at RT. Three 15-min washes between steps were performed with TBS-0.01% Tween 20 (Merck Millipore, Burlington, MA, USA, 8.22184.0500). Bound antibodies were detected with an Odyssey Infrared Imager (LI-COR), and densitometric analysis was performed using the Odyssey V.3 software (LI-COR).

### 2.8. Silencing of CD36 Expression

To silence CD36 expression, undifferentiated THP1 cells were transfected with 10 nM of a set of 4 small-interfering RNAs (siRNAs) targeting CD36, or an equal concentration of a non-targeting negative control pool (ON-TARGET plus siRNA, L-010206–00, and D-001810–10, Thermo Fisher Scientific) using INTERFERin (Polyplus, Illkirch, France, 409), as previously performed [30,31]. After 24 h, the medium was replaced, and cells were differentiated for 24 h in culture medium supplemented with 50 ng/mL of PMA. Next, the PMA-containing medium was replaced with culture medium, and the cells were further incubated for 24 h before being tested for CD36 expression by flow cytometry analysis and used in functional assays. To assay CD36 surface expression, cells were then incubated with 1 mg of moAb anti-CD36 (ImmunoTools GmbH, Friesoythe, Germany) for 90 min at 4 °C in blocking buffer (PBS containing 10% human AB serum, 2% FCS, and 0.02% NaN_3_). Cells were washed once with 3 mL of PBS containing 2% FCS and 0.02% NaN3 (washing buffer), and incubated with fluorescein isothiocyanate-conjugated anti-mouse IgG/IgM antibody (BD Biosciences) in blocking buffer for 45 min at 4 °C. After washing cells with 3 mL of washing buffer, flow cytometric analysis was performed on a BD LSRFortessa instrument using FACSDiva software (BD Biosciences). The integrated mean fluorescence intensity (iMFI) was computed by multiplying the relative frequency (percentage of positive) of cells, expressing each marker with the mean fluorescence intensity (MFI) of the cell population.

### 2.9. Measurement of Cytokine Secretion

THP1 MΦ or MDM (5 × 10^4^ cells/well) were incubated with oxLDL under NG or HG conditions for the indicated times (24 h or 72 h) in culture medium containing 5% FCS. The cells were then stimulated with 10 ng/mL of the TLR4 agonist lipopolysaccharide (LPS, from *E. coli* 0111:B4). After 4 h for TNF or 24 h for IL1B, IL8, IL10, and MCP1, culture supernatant fractions were collected and assayed for cytokine/chemokines by ELISA, following the manufacturer’s instructions (BD Bioscience OptEIA ELISA sets for TNF 55212, IL1B 557953, IL8 555244, and IL10 555157; R&D Systems DuoSet ELISA for MCP1 DY279-5). Color was developed by addition of the 3,3′,5,5′-tetramethylbenzidine liquid substrate (Sigma-Aldrich, T8665), and the optical density was read at 650 nm on a Varioskan Flash microplate reader (Thermo Fisher Scientific Inc.).

### 2.10. Flow Cytometry Analysis of Polarization Markers

Peripheral blood monocytes (10^6^) were incubated for 72 h under NG or HG conditions and treated with oxLDL at a 5% final FCS concentration. INF/LPS (50/100 ng/mL, Preprotech/Sigma-Aldrich, 300-02/14391), IL4 (40 ng/mL, Preprotech, London, UK, 200-04), and IL10 (50 ng/mL, Preprotech, 200-10) were used as controls for macrophage polarization. Cells were detached with accutase (Sigma-Aldrich, A6964), washed twice in PBS, and incubated with 50 μL of PBS containing 10% human AB serum, 2% FCS, and 0.02% NaN_3_ for 30 min on ice. For immunophenotyping, the cells were then stained with a combination of fluorescently conjugated monoclonal antibodies against HLADR, CD80, CD23, CD206, and CD163 (BD Biosciences, 555811, 561135, 564063, 558690, and 563889, respectively) for 20 min in Brilliant Stain buffer (BD Biosciences, 563794). They were then rinsed with washing buffer (PBS containing 2% FCS and 0.02% NaN3) and fixed with 1% paraformaldehyde (PanReac AppliChem, 211511). Flow cytometry analysis was performed on a BD LSRFortessa instrument using FACSDiva software (BD Biosciences). The integrated mean fluorescence intensity (iMFI) was computed by multiplying the relative frequency (percentage of positive) of cells, expressing each marker with the mean fluorescence intensity (MFI) of the cell population.

### 2.11. Immunophenotyping

Immunophenotyping analyses were performed on a total of 53 subjects (25 with T2D and 28 without T2D). Blood samples were collected in the fasting state into vacutainers (BD Bioscience, K2E367525) containing EDTA for anticoagulation, and after a 1 h RT incubation, immunophenotypic analysis of peripheral blood lymphocytes was performed as described [37]. Briefly, 200 μL of whole peripheral blood samples was incubated for 5 min with ammonium chloride lysis reagent (BD Biosciences, 55899) for erythrocyte lysis, followed by washing with PBS and subsequent staining with a combination of fluorescently conjugated monoclonal antibodies against CD4, CD14, CD16, and TLR4 (BD Biosciences 563875, 563743, 563690, and 564215, respectively) for 20 min in Brilliant Stain buffer (BD Biosciences, 563794). Flow cytometry analysis was performed on a BD LSRFortessa instrument using FACSDiva software (BD Biosciences).

### 2.12. sCD14 ELISA

Soluble CD14 analyses were performed on a total of 112 subjects (55 with T2D and 57 without T2D). Plasma concentrations of human sCD14 were measured using the commercially available Human CD14 Quantikine ELISA Kit (DC140, R&D systems). Experiments were performed in duplicate, with appropriate dilutions according to the manufacturer’s instructions. The absorbance was read at 450 nm using a Varioskan Flash microplate reader (Thermo Fisher Scientific). The protein concentration was estimated using a standard curve, based on the standards’ measurements.

### 2.13. Phospho-Proteome Array

The proteome-profiler human phospho-kinase array kit (R&D Systems, ARY003B) was used to assess phospho-kinase activity upon treatment. The human phospho-kinase array is a nitrocellulose membrane spotted with antibodies against 46 kinase phosphorylation sites in duplicate. MDM from n = 2 healthy donors in duplicate were treated with oxLDL for 24 h under NG or HG conditions. Cells were lysed, and 300 μg of the cell lysates were subjected to incubation with membranes overnight at 4 °C according to the manufacturer’s instructions. Briefly, membranes were washed and incubated with a cocktail of phospho-site-specific biotinylated antibodies at RT for 2 h, washed, and then incubated with IRDye^®^ 800CW streptavidin (LI-COR, 926-32230) 1:2000 (*v*:*v*) for 30 min. Images were collected with an Odyssey Infrared Imager (LI-COR), and densitometric analysis was performed using Odyssey V.3 software (LI-COR) after background intensity and control spot correction for each membrane.

### 2.14. Statistical Analysis

Data are presented as the mean ± SEM. Statistical significance was calculated by the Mann–Whitney test (two-tailed) using Prism (GraphPad, GraphPad Software, Boston, MA, USA). Significance was accepted at * *p* < 0.05, ** *p* < 0.01, or *** *p* < 0.001. The numbers of cohorts and n values for each experiment are indicated in the figure legends.

## 3. Results

### 3.1. High Glucose Potentiates Macrophage oxLDL Uptake and Foam Cell Formation through CD36 Upregulation

**Figure 1:**
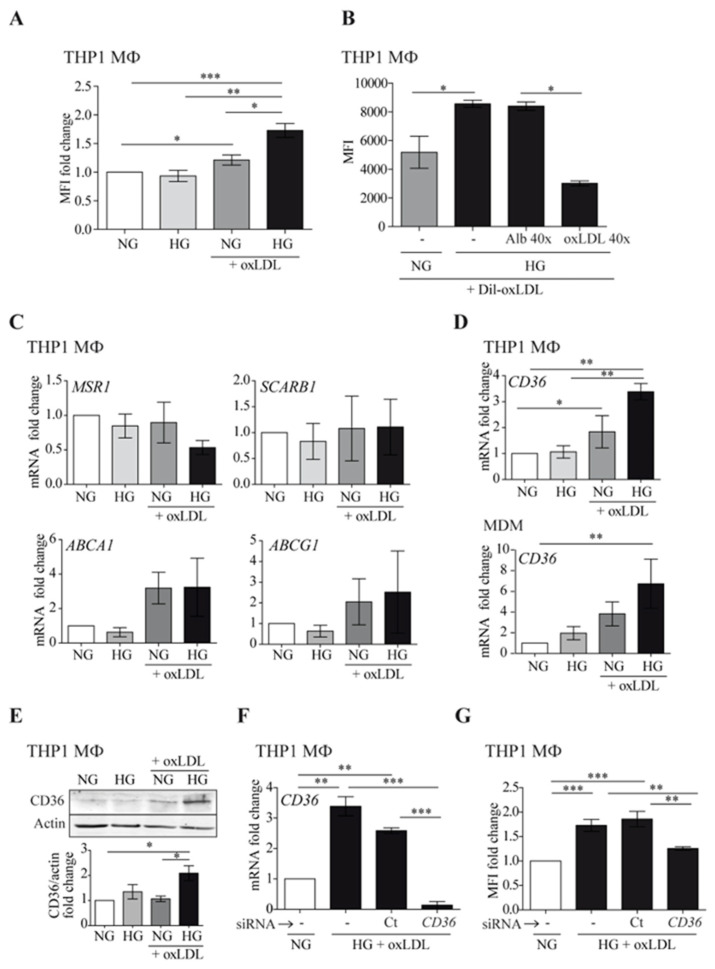
High glucose potentiates macrophage oxLDL uptake through CD36 upregulation. Cells were treated with 150 μg/mL of oxLDL for 24 h in culture medium with 5 mmol/L (normal glucose, NG) or 15 mmol/L (high glucose, HG) of glucose. (**A**) THP1 MΦ were fixed and stained with Nile Red, and the lipid content was analyzed by flow cytometry. Data show the mean fluorescence intensity (MFI) fold change from 5 independent experiments relative to untreated cells (NG) ± SEM. (**B**) THP1 MΦ were incubated for 6 h with fluorescent Dil-oxLDL, and oxLDL uptake was analyzed by flow cytometry. Excess human albumin (Alb 40×) was used as a negative competition control and excess oxLDL as a specific competition control (oxLDL 40×). The graph shows MFI values ± SEM from 5 independent experiments. (**C**,**D**) The amount of mRNA encoding MSR1, SCARB1, ABCA1, ABCG1 (**C**), and CD36 (**D**) was measured by RT-PCR, and the data show the mean fold change relative to untreated cells (NG) ± SEM from at least 3 independent experiments. (**E**) THP1 MΦ were lysed and probed by Western blotting with an antibody specific to CD36. Upper panel: Western blot image of a representative experiment. Lower panel: mean protein signal intensity fold change relative to untreated cells (NG) ± SEM from 3 independent experiments. Equal loading was determined by probing against actin. (**F**,**G**) THP1 cells were left untreated (-), transfected with siRNA targeting CD36 (CD36), or transfected with a non-targeting negative control (Ct), and incubated with oxLDL under HG conditions for 24 h. CD36 expression was analyzed by RT-PCR (**F**) and lipid content by Nile Red staining and flow cytometry (**G**). Mean values ± SEM from at least 3 independent experiments are shown (* *p* < 0.05, ** *p* < 0.01, *** *p* < 0.001, Mann–Whitney test). MDM: monocyte-derived macrophages.

We assessed whether hyperglycemia could alter oxLDL-induced foam cell formation (Figure 1). As shown in Figure 1A, high glucose (HG) conditions increased lipid accumulation in THP1 MΦ, compared to normal glucose (NG) levels. In accordance, uptake of fluorescently labeled oxLDL was enhanced in THP1 MΦ cultured in HG compared with those cultured in NG (Figure 1B). The uptake was specific because effective competition could be achieved with a molar excess of non-labeled oxLDL. Next, we assessed whether HG could influence mRNA expression of cholesterol scavenger receptors A1 and B1 (*MSR1*, *SCARB1*) and ATP-binding cassette transporters A1 and G1 (*ABCA1*, *ABCG1*) by real-time PCR in THP1 MΦ. As observed in Figure 1C, HG did not affect the expression of these receptors. However, HG potentiated oxLDL-mediated enhancement of CD36 in both THP1 MΦ and human monocyte-derived macrophages (MDM) at mRNA and protein levels (Figure 1D,E, respectively). These data suggest that under such conditions, the elevated oxLDL uptake was strongly dependent on CD36 action. To confirm this finding, CD36 expression was silenced using specific siRNA in THP1 MΦ (Figure 1F). Downregulation of CD36 expression concomitantly reduced HG-induced lipid accumulation to the levels observed with NG treatment (Figure 1G). Taken together, these data suggested that HG may potentiate macrophage oxLDL uptake and foam cell formation through CD36 upregulation.

### 3.2. Macrophage Inflammatory Responses to High Glucose Are Potentiated by oxLDL

**Figure 2:**
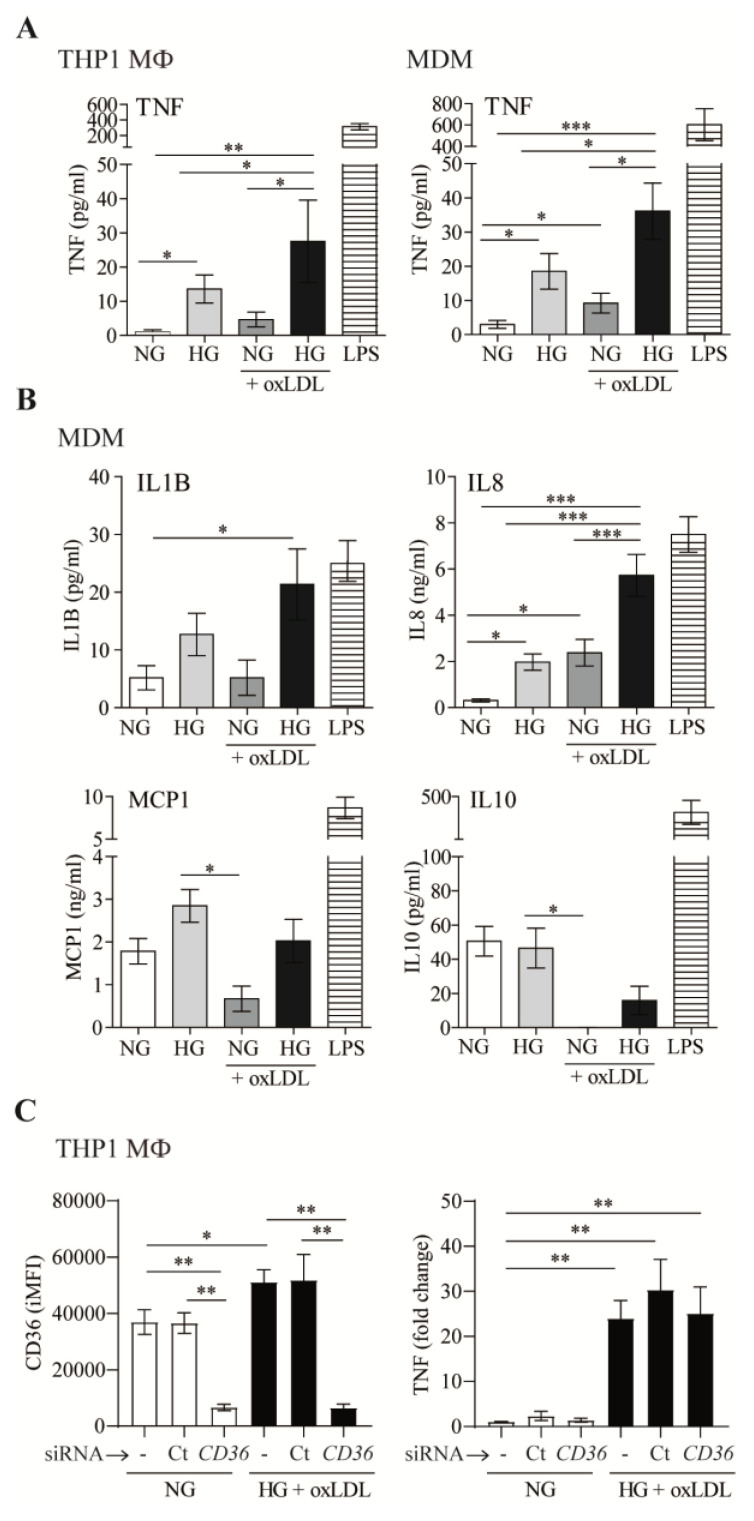
OxLDL increases high-glucose-dependent inflammatory responses in macrophages. (**A**,**B**) THP1 MΦ or MDM were treated with 150 μg/mL of oxLDL for 72 h in culture medium with 5 mmol/L (normal glucose, NG) or 15 mmol/L (high glucose, HG) of glucose. Cells were also stimulated with 10 ng/mL of LPS in NG. Culture supernatants were collected, and the amount of TNF (**A**), IL1B, IL8, MCP1, and IL10 (**B**) was analyzed by ELISA. Data from at least 4 independent experiments for THP1 MΦ or 4 donors for MDM, performed in triplicate, are shown. (**C**) THP1 cells were left untreated (-), transfected with siRNA targeting *CD36* (*CD36*), or transfected with a non-targeting negative control (Ct), incubated with oxLDL under HG conditions for 24 h. CD36 surface expression was analyzed by flow cytometry, and TNF in culture supernatants was measured by ELISA. The graph shows the mean ± SEM from 3 independent experiments performed in triplicate (* *p* < 0.05, ** *p* < 0.01, *** *p* < 0.001, Mann–Whitney test). MDM: monocyte-derived macrophages. iMFI: integrated mean fluorescence intensity.

Next, we examined the role of oxLDL in modulating the cytokine and chemokine secretion of five key mediators of atherosclerotic disease: TNF, IL1B, IL8, MCP1, and IL10 (Figure 2). LPS was used in these assays as a positive control (Figure 2A,B). Our results suggested that HG increases TNF secretion by THP1 MΦ and MDM, which is enhanced by oxLDL (Figure 2A). Similar results were observed by analyzing the amounts of IL1B and IL8 in MDM culture supernatants (Figure 2B). No significant changes were observed for MCP1. In contrast, secretion of the anti-inflammatory cytokine IL10 was almost abrogated by oxLDL, regardless of the amount of glucose in the medium. This is of relevance, since IL10 is an important feedback control mechanism to limit excessive inflammatory responses.

Of note, mannitol (15 mmol/L) was used as an osmotic control in these experiments, and its addition did not modify the levels of any of the cytokines/chemokines analyzed, showing similar results to the control NG. Taken together, these data suggested that the presence of oxLDL potentiates HG-dependent inflammatory responses in macrophages.

Considering that CD36 plays a central role in controlling inflammation induced by oxLDL in macrophages [2], we next assessed whether CD36 could mediate HG-oxLDL-increased TNF secretion. siRNA treatment led to ~85% silencing of CD36 protein surface expression (Figure 2C). However, the downregulation of CD36 expression did not influence the levels of this cytokine in HG + oxLDL conditions (Figure 2C). Therefore, although CD36 plays a key role in lipid accumulation under HG conditions, our data suggested that this transporter does not participate in concomitant macrophage pro-inflammatory responses.

### 3.3. High Glucose and oxLDL Moderately Influenced Macrophage Polarization

**Figure 3:**
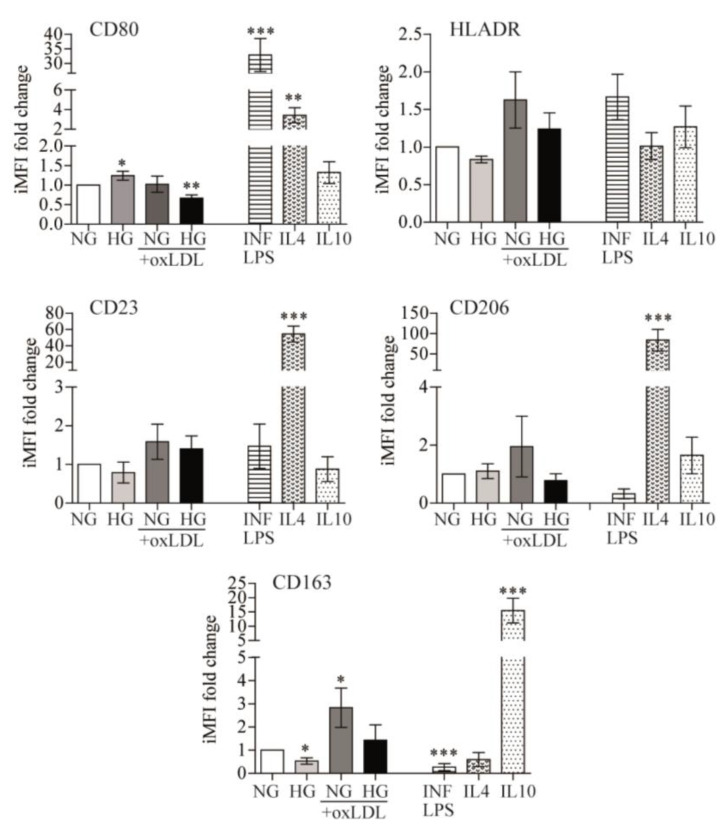
Effects of glucose and oxLDL on macrophage polarization. PB monocytes were incubated for 72 h with 150 μg/mL of oxLDL in culture medium in the presence of 5 mmol/L (NG) or 15 mmol/L (HG) of glucose and INF/LPS, IL4, or IL10 as polarization controls. CD80, HLADR, CD23, CD206, and CD163 surface expression were analyzed by multicolor flow cytometry. Data from 7 blood donors show the iMFI (integrated mean fluorescence intensity) fold change ± SEM relative to untreated PB monocytes (NG) (* *p* < 0.05, ** *p* < 0.01, *** *p* < 0.001, Mann–Whitney test).

In response to changes in the microenvironment, the inherent plasticity of monocytes/macrophages allows the acquisition of a spectrum of polarization states that find their extremes in either pro-inflammatory (M1 or classically activated) or anti-inflammatory (M2 or alternative non-classical activated) responses, which may be appreciated by differential surface membrane marker expression [8]. Therefore, we assessed whether the observed pro-inflammatory responses were reflected in the differential expression of macrophage polarization markers. To achieve this goal, PB monocytes were cultured in the presence of NG or HG alone or in combination with oxLDL, and changes in phenotypic markers of macrophage polarization were compared to well-established inducers of M1 or M2 macrophage states, namely, INF/LPS, IL4, or IL10, respectively (Figure 3).

Flow cytometry analysis of CD80 and HLADR levels (M1 markers), as well as CD23, CD206, and CD163 (M2 markers), revealed that, in comparison to control (NG) cells, HG induced higher CD80 (1.241 ± 0.116-fold, *p* = 0.047) and lower CD163 expression (−2.328 ± 0.705-fold, *p* = 0.0313), oxLDL + NG increased CD163 expression (2.967 ± 0.8474-fold, *p* = 0.0386), and oxLDL + HG combination treatment reduced expression of the M1 marker CD80 (−1.651 ± 0.2814-fold, *p* = 0.0026) (Figure 3). These data suggest that HG marginally switched macrophages toward an M1 phenotype, while oxLDL drove them toward an M2 phenotype. Whereas, in combination, HG seems to reduce oxLDL-related effects. Interestingly, these variations were far lower than those induced by INF/LPS, IL4, or IL10. Overall, the data suggested that polarization may not be the main effector by which HG, alone or combined with oxLDL, alters macrophage inflammatory responses.

### 3.4. Effects of OxLDL and High Glucose on TLR4 and CD14 mRNA and Protein Expression

**Figure 4:**
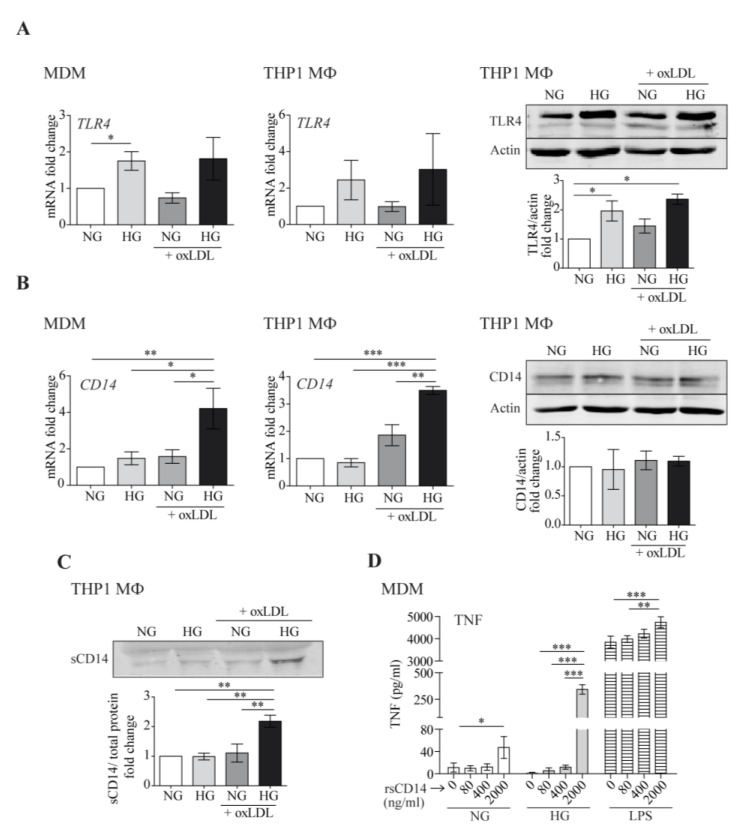
High glucose and oxLDL modify TLR4 and CD14 expression and promote sCD14 shedding. MDM or THP1 MΦ were treated with 150 μg/mL of oxLDL for 24 h in culture medium with glucose concentrations of 5 mmol/L (normal glucose, NG) or 15 mmol/L (high glucose, HG). TLR4 (**A**) and CD14 (**B**) mRNA levels were analyzed by RT-PCR (left) and protein levels by Western blotting (right). Panels: representative Western blot images, graphs: mean ± SEM fold induction levels relative to untreated cells (NG). (**C**) The culture supernatants were TCA-precipitated and probed by Western blotting against CD14. Upper panel: representative Western blot image. Lower graph: band intensity values vs. total protein. Fold induction levels are relative to untreated cells (NG). Data from 6 blood donors for MDMs or 3 independent experiments for THP1 MΦ are shown. (**D**) MDMs were treated with the indicated amounts of rsCD14 in culture medium containing NG, HG, or LPS (10 ng/mL) for 4 h, and the amount of TNF in the culture supernatants was measured by ELISA. The graph shows the mean ± SEM from 3 independent experiments performed in triplicate (* *p* < 0.05, ** *p* < 0.01, *** *p* < 0.001, Mann–Whitney test). MDM: monocyte-derived macrophages.

TLR4/CD14 co-receptors mediate oxLDL-induced inflammation [38]. Moreover, HG induces TLR4 expression and activity in monocytes, resulting in a pro-inflammatory response [24,39]. In accordance, we observed that exposure to HG enhanced TLR4 mRNA and protein expression in human MDM as well as THP1 MΦ (Figure 4A). However, such HG-mediated elevation of TLR4 was not further increased by the addition of oxLDL, suggesting that TLR4 upregulation was not a main determinant of the enhanced inflammatory responses. More interestingly, HG and oxLDL increased mRNA levels of CD14 in both macrophage models (Figure 4B, left and center). However, the cellular CD14 protein levels were not concomitantly elevated (Figure 4B, right), possibly suggesting increased CD14 shedding under HG + oxLDL conditions.

Western blotting of THP1 culture supernatants showed that the HG + oxLDL treatment almost doubled sCD14 secretion compared with individual stimulus treatment or the medium alone (Figure 4C). As sCD14 stimulates pro-inflammatory cytokine/chemokine production in macrophages in an independent manner or in combination with other inflammatory stimuli such as LPS [40], we assessed its pro-inflammatory abilities under HG. To achieve this goal, MDM were treated with increasing concentrations of human recombinant sCD14 (rsCD14) under NG, HG, or in combination with LPS as a positive control for the synergistic response. As shown in Figure 4D, the addition of rsCD14 to macrophages induced TNF secretion under NG conditions (up to ~5-fold). Interestingly, this induction was more evident under HG conditions (up to ~250-fold), suggesting that sCD14 could potentiate macrophage inflammatory responses under HG.

### 3.5. High Glucose + oxLDL-Mediated Induction of CD14 and Inflammatory Responses Are PRAS40/Akt-Dependent

**Figure 5:**
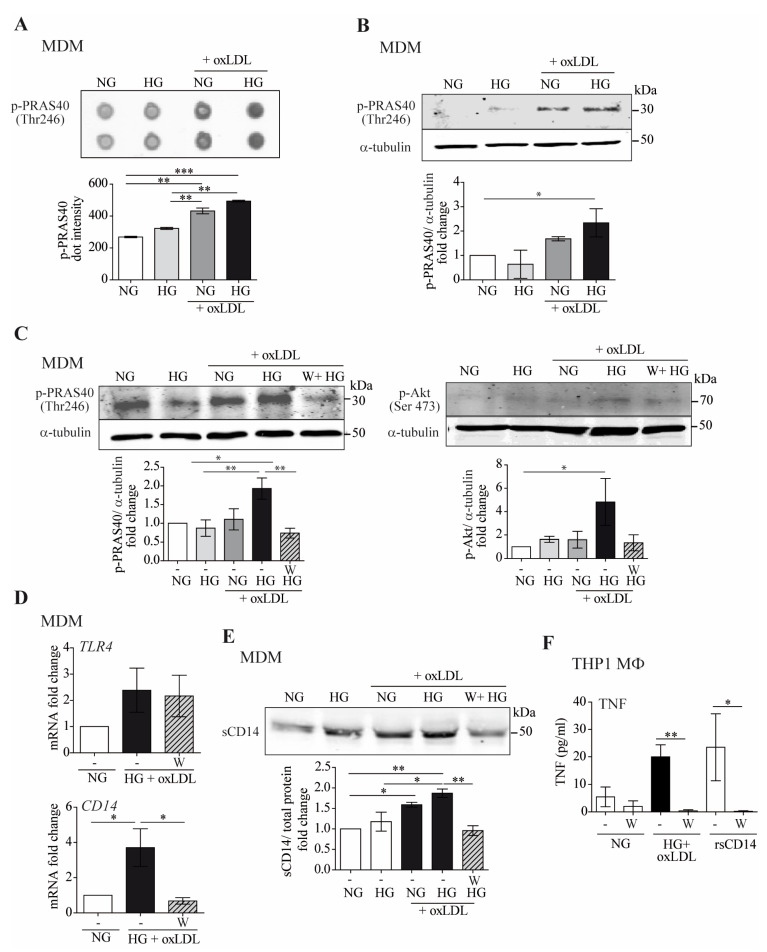
The combination of high glucose and oxLDL promotes sCD14 shedding though Akt-PRAS40 pathway activation. MDM were treated with 150 μg/mL of oxLDL for 24 h in culture medium containing 5 mM (normal glucose, NG) or 15 mM (high glucose, HG) of glucose. (**A**) MDM lysates were exposed to phospho-kinase array spotted membranes. Upper panel: dot images. Lower graph: quantification of dot intensities. (**B**) Cell lysates were probed with anti-pPRAS40 (Thr246) by Western blotting, and equal loading was determined by probing with α-tubulin. Upper panel: representative Western blot images, lower panel: mean ± SEM fold induction levels relative to untreated MDMs (NG) from 2 independent donors. (**C**–**F**) Cells were incubated for 45 min with 10 µM of wortmannin (W) for 24 h prior to oxLDL stimulation under NG or HG conditions. (**C**) PRAS40 (Thr246) and Akt (Ser473) phosphorylation determined by Western blotting. Upper panel: representative images. Lower panel: graph showing fold induction levels relative to untreated cells (NG) from 4 donors. (**D**) TLR4 and CD14 mRNA expression analyzed by RT-PCR. The data show the mean fold change relative to untreated cells (NG) ± SEM from 4 donors. (**E**) sCD14 in culture supernatants measured by Western blotting. Upper panel: representative Western blot images. Lower panel: band intensity values/total protein. Fold induction levels relative to untreated cells (NG) from 4 donors are shown. (**F**) THP1 MΦ were pre-incubated with wortmannin (W) and then treated with NG, HG + oxLDL, and with 2 µg/mL of rsCD14. After 24 h, TNF in culture supernatants was measured by ELISA. Graph showing the mean ± SEM from 3 independent experiments performed in triplicate (* *p* < 0.05, ** *p* < 0.01, *** *p* < 0.001, Mann–Whitney test). MDM: monocyte-derived macrophages.

Next, we conducted a human phospho-kinase array to gain further insights into the cellular mechanisms governing macrophage responses that distinguish individual HG or oxLDL vs. their combination. We screened MDM-treated lysates in a phosphorylation array for simultaneous detection of 43 kinase phosphorylation sites (Figure 5).

These assays revealed the proline-rich Akt substrate of 40 kDa (PRAS40) as a putative candidate because it showed high phosphorylation levels under HG + oxLDL (Figure 5A). Similar results were observed by Western blot analysis of cell lysates from two independent donors (Figure 5B). PRAS40 acts at the intersection of the protein kinase B (Akt) and mammalian target of rapamycin (mTOR)-mediated signaling pathways [41].

To assay the effects of HG + oxLDL in this pathway, we pharmacologically blocked the PI3K/Akt/mTOR pathway with wortmannin (W) and assayed PRAS40 and upstream Akt phosphorylation. Western blot experiments showed that HG + oxLDL-dependent PRAS40 and Akt phosphorylation were abrogated when cells were preincubated with wortmannin, thus suggesting that oxLDL + HG could activate the Akt/PRAS40 pathway (Figure 5C). Next, we assayed the involvement of the Akt/PRAS40 pathway in HG + oxLDL-dependent inflammatory responses. First, we observed that pre-incubation with wortmannin had no effect on HG-induced TLR4 expression (Figure 5D, top). More interestingly, HG + oxLDL-dependent CD14 mRNA upregulation (Figure 5D, bottom) and sCD14 protein secretion (Figure 5E) did not occur when the pathway was blocked by the addition of wortmannin. In agreement with this finding, the inflammatory response induced by HG + oxLDL or sCD14 was also abrogated by pharmacological inhibition of the Akt pathway (Figure 5F). Taken together, these results suggest that Akt/PRAS40 is involved in sCD14 secretion and induction of inflammatory responses in macrophages under hyperglycemic and proatherogenic conditions.

### 3.6. TLR4 Is Elevated in a Subset of Non-Classical Monocytes from Subjects with T2D and Atherosclerosis

**Figure 6:**
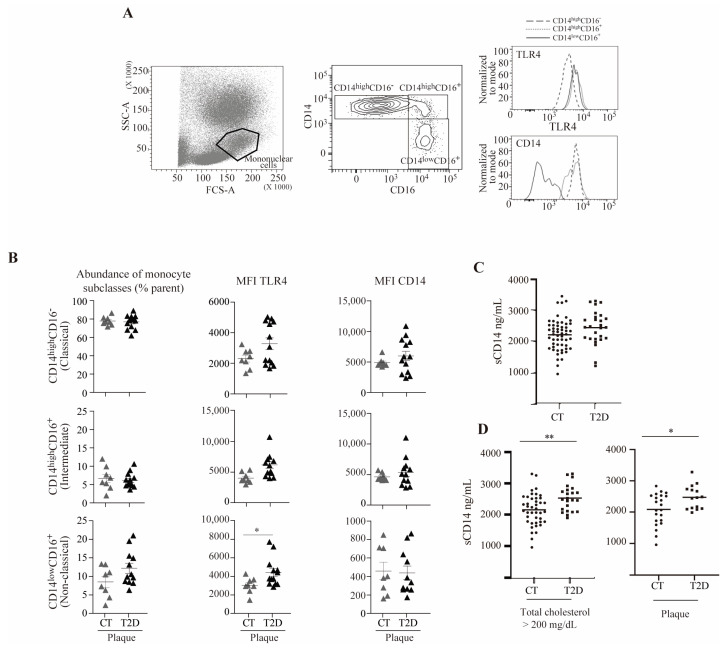
TLR4 and sCD14 are elevated in a subset of non-classical monocytes from subjects with T2D and atherosclerosis and in T2D subjects with hypercholesterolemia or atherosclerosis, respectively. (**A**) Representative plots of the total blood flow cytometry gating strategy: (left) forward scatter (FCS-A) and side scatter (SSC-A) showing the mononuclear cell population, (center) monocyte expression pattern of CD14 and CD16 showing the classical (CD14^high^CD16^−^), intermediate (CD14^high^CD16^+^), and non-classical (CD14^low^CD16^+^) subclasses, and (right) TLR4 and CD14 fluorescence histograms. (**B**) Abundance of monocyte subclasses, and TLR4 and CD14 mean fluorescence intensity (MFI) in the control (CT) and diabetic group (T2D) in the presence of subclinical atherosclerosis (plaque). (**C**) sCD14 plasma levels as measured by ELISA from subjects with and without diabetes. (**D**) sCD14 plasma levels as measured by ELISA from subjects with hypercholesterolemia (total cholesterol > 200 mg/dL) with and without diabetes (left) and from subjects with atherosclerosis with and without diabetes (right) (* *p* < 0.05, ** *p* < 0.01, Mann–Whitney test).

To determine whether our observations could reflect the state of in vivo circulating monocytes, we next evaluated the cell surface expression of TLR4 and CD14 co-receptors in PBMC from 28 subjects without diabetes and 25 subjects with T2D, as well as their association with subclinical atherosclerosis (Figure 6).

As shown in Figure 6A, the mononuclear cell population was gated based on forward scatter (FCS-A)/side scatter (SSC-A), and monocytes were defined as single live cells within the mononuclear cells. Three monocyte subsets, classical, intermediate, and non-classical, were determined based on their CD14/CD16 expression (CD14^high^CD16^−^, CD14^high^CD16^+^, and CD14^low^CD16^+^, respectively), and the mean fluorescence intensity (MFI) of TLR4 and CD14 in the three monocyte subsets was measured. The analyses were performed in subjects with subclinical atherosclerotic plaques with or without T2D (Figure 6B). Our results suggested that no significant changes were observed in the proportion of monocyte subclasses between subjects with or without T2D. In contrast, in subjects with subclinical atherosclerotic plaques, the expression of cell surface TLR4 in the non-classical monocytes’ subset was higher in subjects with T2D compared to control subjects. In these analyses, no differences were observed in CD14 surface levels between subjects with or without T2D.

### 3.7. sCD14 Is Elevated in T2D Subjects with Hypercholesterolemia or Atherosclerosis

Circulating concentrations of sCD14 in serum of subjects with T2D (n = 69) did not differ from subjects without diabetes (n = 70) (Figure 6C). It is noteworthy that sCD14 concentrations were significantly higher in subjects with diabetes and hypercholesterolemia (total cholesterol higher than 200 mg/dL) compared to subjects without diabetes with hypercholesterolemia. Moreover, sCD14 concentrations were significantly higher in subjects with diabetes with atherosclerotic plaques compared to subjects without diabetes and without atherosclerotic plaques, possibly reinforcing the notion that CD14 may be released under these settings (Figure 6D).

## 4. Discussion

Increased serum concentrations of oxLDL is a common trait in diabetic subjects [42]; however, the mechanistic action of the combined impact of these two insults is poorly defined. In this study, we examined the inflammatory effect of high glucose concentrations in combination with increased oxLDL on human macrophages. Our data confirmed previous data [43], whereby the coincubation of macrophages with HG + oxLDL promoted CD36-dependent foam cell formation. Our data also confirmed that inflammation was enhanced by HG [44]. Remarkably, in our study, the pro-inflammatory effect was potentiated by treatment with oxLDL, suggesting an increased pro-inflammatory response by macrophages activated by combined pro-atherogenic insults. From a translational point of view, our data may at least partly explain the pathogenesis of the accelerated atherosclerosis that accompanies diabetes.

Regarding macrophage polarization, our results suggested that polarization may not be the main effector by which HG, alone or combined with oxLDL, alters macrophage inflammatory responses. These experiments were performed on macrophages grown in the absence of any growth or activating factor (i.e., M0 macrophages). In this regard, atherosclerotic plaques contain macrophages with many phenotypes [45]. Since these are plastic cells, it would be interesting, in future studies, to determine the contribution of oxLDL and glucose in reprograming the different phenotypes.

In our search for mechanisms to explain the increased pro-inflammatory responses in combined hyperglycemic and pro-atherogenic conditions, we focused our attention on the role of CD36, TLR4, and CD14 in the pro-inflammatory response to either oxLDL [2,46] or high glucose [24,47], as well as their combined contribution (i.e., oxLDL + HG), which had not previously been addressed. In these settings, our results suggested that CD36 played a key role in macrophage lipid accumulation, as it has been well-established [48]. However, this molecule did not appear to participate in the induction of concomitant macrophage pro-inflammatory TNF responses. Regarding the TLR4 receptor, considering that it mediates inflammation under HG [24,47], we postulated that its over-expression could be the cause of the exacerbated inflammation in combination with oxLDL. However, HG-induced TLR4 expression was not further increased by pro-atherogenic conditions in vitro in primary macrophages from non-diabetic donors. In contrast, the mRNA expression levels of the TLR4 co-receptor CD14 were induced upon oxLDL + HG treatment in vitro.

CD14 exists as a 55 kDa membrane-associated glycosylphosphatidylinositol (GPI)-anchored form (mCD14) that is present on the surface of myeloid cells, or as a 48/56 kDa soluble form (sCD14) that is produced either by proteolytic cleavage or by secretion without the GPI moiety [49,50]. Our results suggest, for the first time, that HG + oxLDL conditions not only enhance macrophage CD14 mRNA expression, but also induce the release of sCD14, which may significantly contribute to the exacerbated pro-inflammatory cytokine production. sCD14 has been previously reported to stimulate pro-inflammatory cytokine/chemokine production in macrophages and endothelial cells in an independent manner or in combination with other pro-inflammatory stimuli, such as LPS [40,51]. In addition, our data revealed for the first time that under hyperglycemic and pro-atherogenic conditions, the PRAS40/Akt pathway is also involved in a novel mechanism of macrophage shedding of sCD14.

Enhanced sCD14 release may contribute to the pathogenesis of diabetes-related cardiometabolic complications [52]. In line with this, sCD14 plasma levels are elevated under different inflammatory conditions, including atherosclerosis [53], and circulating sCD14 levels are associated with both subclinical vascular disease and with the risk of future clinical cardiovascular disease in older adults [54]. In the carotid artery wall of human atherosclerotic plaques, immunohistochemistry staining showed that CD14 expression correlated with CD68-positive macrophages and associated with complicated lesions [55]. Although we did not directly determine the serum concentration of oxLDL in our group of subjects with diabetes, they were diagnosed for atherosclerosis, and therefore considered as an opportunity to directly test the hypothesis that circulating sCD14 was linked to atherosclerotic plaque formation in a real practice scenario. Accordingly, our data revealed an increase of circulating sCD14 levels in the T2D subjects with subclinical atherosclerosis. However, further studies are needed to assess whether systemic sCD14 levels may predict future cardiovascular complications in subjects with T2D or whether the vascular content of sCD14 is elevated in atherosclerotic lesions of diabetic subjects.

Beyond the role of tissue macrophages, the recruitment of circulating monocytes is also enhanced in inflamed tissues; moreover, monocytes are also a potential source of sCD14 [56]. In this regard, different subsets of circulating monocytes have been identified based on the surface expression of the CD14 and CD16 markers (classical CD14^high^CD16^−^, intermediate CD14^high^CD16^+^, and non-classical CD14^low^CD16^+^), which may be functionally involved in inflammatory vs. repairing actions [43]. Moreover, compiled evidence supports the notion of alterations in circulating monocytes in atherosclerosis [57,58,59]. In our group of subjects, the relative abundance of peripheral blood monocyte subpopulations did not differ between the non-diabetic and T2D subjects. However, larger cohorts should be examined to draw definitive conclusions. Indeed, a relevant flaw of the current study includes the relatively small number of subjects, particularly in the analysis of sCD14 concentrations and immunophenotyping. This, especially in the case of immunophenotyping analysis, may limit the interpretation of the current findings that should be considered with caution. Thus, the current results should be confirmed in future studies with a larger number of subjects. Overall, our data suggested that macrophage polarization or the relative circulating amounts of monocyte subsets is not influenced by HG, neither alone nor in combination with oxLDL.

## 5. Conclusions

In conclusion, our data revealed for the first time, to our knowledge, that sCD14 release is enhanced under HG + oxLDL conditions in vitro, becoming an additional trigger of inflammation. Remarkably, combined HG + oxLDL induction of macrophage inflammation strongly depended on the PRAS40/Akt signaling. The present work may contribute to understanding the mechanisms underlying increased inflammation in diabetes-accelerated atherosclerosis. Moreover, our data unveiled PRAS40/Akt signaling as a potential therapeutic target and for the future development of mechanism-based therapies that target inflammation in arterial wall cells from T2D subjects.

## Figures and Tables

**Table 1:** Clinical characteristics of the study group subjects.

Characteristics	Non-Diabetic Subjects	T2D	*p*-Value
	N = 70	N = 69	
Sex, men	39 (55.7%)	34 (49.3%)	0.555
Age, years	56.5 [47.0; 62.8]	63.0 [56.0; 69.0]	<0.001
Hypertension	15 (21.4%)	47 (68.1%)	<0.001
Dyslipidemia	20 (28.6%)		<0.001
BMI, kg/m^2^	25.6 [23.9; 27.7]	28.9 [26.6; 32.0]	<0.001
Tobacco exp	35 (50.7%)	36 (52.2%)	0.931
Glucose, mg/dL	92.0 [85.0; 98.0]	147 [120; 206]	<0.001
Triglycerides, mg/dL	89.0 [74.0; 128]	128 [97.0; 168]	<0.001
Total cholesterol, mg/dL	213 [189; 234]	202 [148; 220]	0.024
HDL cholesterol, mg/dL	56.4 [48.1; 66.5]	45.0 [38.2; 57.0]	<0.001
LDL cholesterol, mg/dL	121 [94.0; 152]	93.4 [60.2; 138]	0.031
HbA1c, %	5.60 [5.40; 5.90]	7.80 [6.80; 8.75]	<0.001
Plaque, n (%)	38 (54.3%)	44 (63.8%)	0.335

**Table 2:** Primers used in this study.

Gene	Forward Primer 5′ --> 3′	Reverse Primer 5′ --> 3′	Tm °C
*CD36*	GAGAACTGTTATGGGGCTAT	TTCAACTGGAGAGGCAAAGG	59.8/63.1
*SRA1*	CCAGGGACATGGGAATGCAA	CCAGTGGGACCTCGATCTCC	67.5/66.6
*SCARB1*	TCAGCTCCCAAGGGCTCTGTGC	AAAGGCGCTTTGCCTGGCCT	71.6/70.9
*ABCA1*	TGAGCTACCCACCCTATGAACA	CCCCTGAACCCAAGGAAGTG	65.9/65.6
*ABCG1*	CCTGCTGTACTTGGGGATCGGGAACG	CCAGCGCGGCAAACAGCACAAAG	73.0/72.2
*TLR4*	GCTCGGTCAGACGGTGATAG	TAGGAACCACCTCCACGCAG	64.8/66.7
*CD14*	GCTGTGTAGGAAAGAAGCTA	TTTAGAAACGGCTCTAGGTTG	60.2/60.8
*GAPDH*	TCTTCTTTTGCGTCGCCAG	AGCCCCAGCCTTCTCCA	63.8/66.0

## Data Availability

The data presented in this study are available upon request from the corresponding author.
